# Influences of LIN-12/Notch and POP-1/TCF on the Robustness of Ventral Uterine Cell Fate Specification in *Caenorhabditis elegans* Gonadogenesis

**DOI:** 10.1534/g3.115.022608

**Published:** 2015-10-14

**Authors:** Maria D. Sallee, Taner Aydin, Iva Greenwald

**Affiliations:** * Department of Genetics and Development, Columbia University Medical Center, New York 10032; † Department of Biochemistry and Molecular Biophysics, Columbia University Medical Center, New York 10032

**Keywords:** *C. elegans*, Notch, POP-1, gonadogenesis, *lin-12*

## Abstract

The prospective ventral uterus of the hermaphrodite gonad primordium consists of two pairs of sister cells, with each pair consisting of a proximal “α” cell and a distal “β” cell. All four cells initially are competent to become the anchor cell (AC), a unique cell type that acts as the organizer of subsequent uterine and vulval development. However, the β cells soon lose this competence and always become ventral uterine precursor cells (VUs), whereas the α cells maintain their AC competence longer, until *lin-12*/Notch-mediated interactions between them specify one as the AC and the other as a VU. Here, we investigate this asymmetry in developmental potential and VU fate specification between the α and β sister cells. We find evidence that *lin-12* activity contributes to the robustness of βVU fate at elevated temperature, that the *Caenorhabditis elegans Notch* paralog *glp-1* is not functionally redundant with *lin-12* in specifying βVU fate, and that the activity of POP-1, the sole *C. elegans* TCF ortholog, influences βVU fate. We propose a model for how Wnt and LIN-12/Notch signaling together lead to robust specification of the βVU fate.

Kimble and Hirsh (1979) described the cell lineage and morphogenetic events of early hermaphrodite gonadogenesis. When the L1 larva hatches, the gonad primordium contains four cells: the somatic progenitors Z1 and Z4 and the germline progenitors Z2 and Z3. During the middle of the first larval (L1) stage, Z1 and Z4 begin their mirror-image lineages to generate 12 descendants by the early L2 stage (Figure 1A). By the late L2 stage, these 12 cells, which until then are intermingled with germline stem cells, rearrange to form the somatic gonad primordium: most somatic cells coalesce in the proximal region, with the distal tip cells (DTCs) remaining at the distal ends to nurture the germline and lead the extension of the gonad arms.

**Figure 1:**
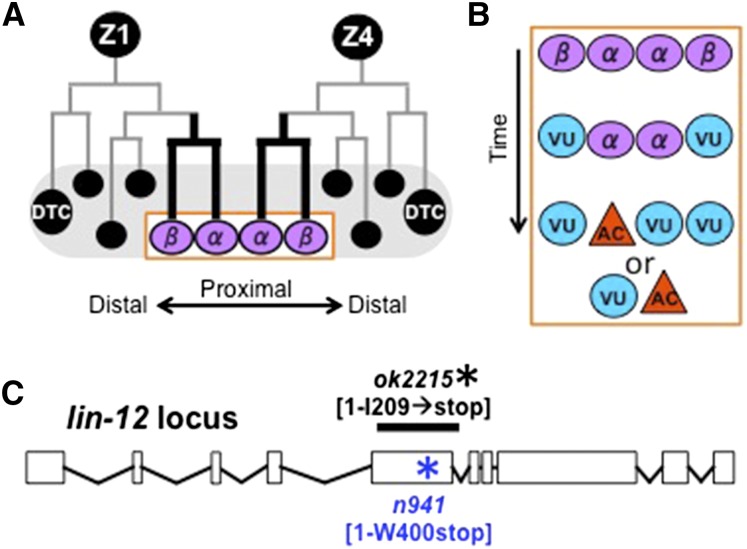
Somatic gonad formation and *lin-12*−null alleles. (A) The somatic gonad founder cells Z1 and Z4 begin the first phase of their lineage in the mid L1 stage and the resulting descendants form the somatic gonad primordium in the L2 stage (Kimble and Hirsh1979). The DTCs indicate the distal axes; the ventral uterus is proximal. The α and β cells give rise to the ventral uterus and initially have the potential to become an anchor cell (AC). (B) Specification of α and β cells over time. The β cells soon lose competence to be the AC and always become ventral uterine precursor cells (VUs); the α cells maintain their AC competence longer, until *lin-12*/Notch-mediated interactions between them specify one as the AC and the other as a VU (Seydoux and Greenwald 1989; Seydoux *et al.* 1990). Half the time, the α cell derived from Z1 (Z1.ppp) becomes the AC and half the time, the α cell derived from Z4 (Z4.aaa) becomes the AC (Kimble and Hirsh 1979). (C) The *lin-12* locus, showing the position of the *n941* point mutation associated with a stop codon and the *ok2215* deletion mutation, which causes a frameshift after amino acid I209 until a stop codon occurs about 80 amino acids later.

The ventral uterine region of the somatic primordium contains three ventral uterine precursor cells (VUs), blast cells that later generate descendants that form the ventral uterus; and an anchor cell (AC), a specialized, terminally differentiated cell that serves as a signaling hub to organize subsequent uterine and vulval development. These cells are formed when two parent cells, Z1.pp and Z4.aa, each yield a proximal “α” and a distal “β” cell daughter (Figure 1B). Laser ablation experiments indicate that the α and β cells initially are competent to become an AC, but the β cells soon lose this competence and always become VUs (Seydoux *et al.* 1990), here called “βVUs.” The two α cells maintain their competence longer than the β cells, and their fates are resolved through LIN-12/Notch-mediated interactions between them, the “AC/VU decision”: one becomes the AC and the other a VU (Seydoux and Greenwald 1989; Wilkinson *et al.* 1994), here called the “αVU.” Thus, in the absence of *lin-12* activity, as in a *lin-12(0)* [null] mutant (Figure 1C), both α cells become ACs [(Greenwald *et al.* 1983); reviewed in (Greenwald 2012)], but most of the β cells still become βVUs, indicating that *lin-12* is not required for the βVU fate. However, *lin-12* influences the fate of the β cells: in a wild-type hermaphrodite, a β cell can form an AC if both α cells are ablated before somatic primordium formation, but in *lin-12(0)* hermaphrodites, a β cell can become an AC even if the ACs are ablated after somatic primordium formation. Thus, *lin-12* activity is not necessary in β cells to specify a VU fate, although it enhances the potential to choose the VU fate.

In this study, we investigate the role of *lin-12* and the asymmetry in developmental potential and VU fate specification between the α and β sister cells. We show that the *lin-12*−null phenotype is heat-sensitive and that the *lin-12*−independent βVU fate does not reflect activity of *glp-1*, the other *Caenorhabditis elegans Notch* gene. Instead, we provide evidence that POP-1, the sole *C. elegans* TCF ortholog, influences βVU fate. Our observations suggest a model in which Wnt and LIN-12/Notch signaling together lead to robust specification of the βVU fate.

## Materials and Methods

### Alleles and transgenes

The *C. elegans* Bristol strain N2 was used as the wild type in this study. The following LGIII mutations were used: *pha-1(e2123ts)*, *lin-12(n941)*, *lin-12(ok2215)*, and *glp-1(q46)*. The *lin-12(ok2215)* allele encodes a 1227-bp deletion in exon 5 and intron 5 and is a predicted null allele based on the early stop codon generated as a result of the deletion; the phenotypic characterization herein is consistent with that prediction. *hT2[qIs48]* is a translocation [*hT2*] marked with *myo-2*p::GFP that balances the *lin-12* region of LGIII (Siegfried and Kimble 2002).

The following transgenes were used as markers or for experimental manipulations:

***arIs51*** IV [*cdh-3*p::GFP] (Karp and Greenwald 2003) and ***arIs222*** V [*lag-2*p::2xNLS-TagRFP] (Sallee and Greenwald 2015), both transcriptional reporters, mark the AC.
***arTi3*** V [*hlh-2*prox::GFP-HLH-2] (Sallee and Greenwald 2015) is a single-copy, miniMos-based insertion (Frøkjær-Jensen *et al.* 2014). *hlh-2*prox is a fragment of the *hlh-2* promoter that drives expression in the α and β cells, their parents, and the differentiated AC and VUs (Sallee and Greenwald 2015). However, GFP-HLH-2 is degraded in VUs, so this transgene serves as an AC marker.
***arIs208*** X [*hlh-2*(-5253-1)p::2xNLS-TagRFP] is strongly expressed in the α and β cells and was a convenient red marker for these cells when used in conjunction with *qIs74* for assessing GFP-POP-1 accumulation.
***arEx576*** [*lin-12(+)*, *unc-122*p::GFP, *mir-84*p::2xNLS-YFP] and ***arEx1442*** [*lin-12(+)*, *myo-3*p::mCherry] rescue *lin-12(0)* alleles and were used for mosaic analysis or maintaining *lin-12(0)*-containing strains.
***arEx1552*** and ***arEx1553*** contain the GLP-1-GFP fosmid fosMS4, encoding a protein with GFP inserted in frame in the intracellular domain (see *Transgene generation and analysis*), are expressed in the soma but not in the germline. ***tnIs39***, a gift of David Greenstein and Tim Schedl, is a rescuing transgene that is expressed in the germline. GFP expression was not evident in the L2 stage in the somatic gonad for any of these transgenes.
***qIs74*** [GFP-POP-1] (Siegfried *et al.* 2004) has been used in many studies as the canonical POP-1 reporter.
***arEx2219*** and ***arEx2220*** [*hlh-2*prox::GFP-POP-1] express GFP-POP-1 specifically in the ventral uterus, and ***arEx2222*** and ***arEx2223*** [*hlh-2*prox::GFP] are transcriptional reporters prepared under the same conditions and analyzed in parallel as controls.

The strains used in this study are listed in Table 1.

**Table 1:** Strains used in this study

Figure	Strain Name	Genotype
	GS6259	*lin-12(n941)/hT2(qIs48 [myo-2*p::GFP*])*; *arIs51 [cdh-3*p::GFP*]*
2	GS7747	*lin-12(n941)*; *arTi3 [hlh-2*prox::GFP-HLH-2*]*; *arEx576*
2	GS6123	*lin-12(n941)*; *arIs51*; *arEx576[lin-12(+)]*
2	GS8010	*lin-12(ok2215)*; *arTi3*; *arEx576*
2	GS8009	*lin-12(ok2215)*; *arIs51*; *arEx576*
3	GS6433	*pha-1(e2123)*; *arEx1552 [*GLP-1-GFP fosmid*]*
	GS6434	*pha-1(e2123)*; *arEx1553 [*GLP-1-GFP fosmid*]*
	BS4048	*glp-1(q175)*; *tnIs39 [*GLP-1-GFP fosmid*]*
3D	GS6355	*lin-12(n941) glp-1(q46)/hT2(qIs48)*; *arIs51*; *arEx576*
4A	GS6911	*qIs74 [*GFP-POP-1*]*; *arIs208 [hlh-2*(5.2kb)p::2xNLS-TagRFP*]*
4B	GS7741	*pha-1(e2123ts)*; *arIs222 [lag-2*p::2xNLS-TagRFP*]*; *arEx2219 [hlh-2*prox::GFP-POP-1*]*
4B (photo)	GS7742	*pha-1(e2123ts)*; *arIs222*; *arEx2220 [hlh-2*prox::GFP-POP-1*]*
4B	GS7744	*pha-1(e2123ts)*; *arIs222*; *arEx2222 [hlh-2*prox::GFP*]*
4B	GS7745	*pha-1(e2123ts)*; *arIs222*; *arEx2223 [hlh-2*prox::GFP*]*
4C, 4D	GS8015	*lin-12(n941) pha-1(e2123ts)*; *arIs222*; *arEx1442 [lin-12(+)]*; *arEx2219*
4C	GS8016	*lin-12(n941) pha-1(e2123ts)*; *arIs222*; *arEx1442*; *arEx2220*
4C	GS8017	*lin-12(n941) pha-1(e2123ts)*; *arIs222*; *arEx1442*; *arEx2222*
4C	GS8018	*lin-12(n941) pha-1(e2123ts)*; *arIs222*; *arEx1442*; *arEx2223*

### Fosmid recombineering and plasmid construction

fosMS4 is a *glp-1* fosmid with *gfp* inserted inside the *glp-1* coding region, replacing amino acids N1193-R1209 to make a GLP-1-GFP fusion protein similar to the well-characterized, rescuing LIN-12-GFP transgene (Levitan and Greenwald 1998). Recombineering to generate fosMS4 was done essentially as described (Tursun *et al.* 2009) with the *glp-1* fosmid WRM066aC10 and the plasmid pBALU9.

*hlh-2*prox::*gfp-pop-1* was made by amplifying, via polymerase chain reaction, *pop-1* cDNA from pMM414 (Maduro *et al.* 2002), fusing it with *gfp*, and inserting it into pMS155 to make pTA1.
*hlh-2*p::2x*nls-tagRFP* was made by subcloning 2x*nls-tagRFP* into pMS2 (Sallee and Greenwald 2015), replacing the *gfp* and making *hlh-2*[-5253-1]p::2x*nls-tagRFP*::*unc-54* 3′UTR (pMS66).

### Transgene generation and analysis

The GLP-1-GFP fosmid fosMS4 was linearized and injected at 15 ng/μL, along with pBX (*pha-1(+)*) and pCW2.1 (*ceh-22*p::*gfp*) at 1 ng/μL and *Pvu*II-digested N2 gDNA at 50 ng/μL, into 20 *pha-1(e2123ts)* P0 hermaphrodites. *hlh-2*prox-driven transgenes were linearized and injected at 1 ng/μL, along with pBX (*pha-1(+)*) and pCW2.1 (*ceh-22p*::*gfp*) at 1 ng/μL and *Pvu*II-digested N2 gDNA at 50 ng/μL, into 20 *pha-1(e2123ts)*; *arIs222* [*lag-2*p::2xNLS-TagRFP] P0 hermaphrodites. Injected P0s were kept at 15° for 3 d, then shifted to 22° for 4 d. Independent transgenic lines were isolated from F2s, generating a maximum of one line per injected P0. Worms were mounted on 2% agarose pads on a slide and immobilized with 10 mM levamisole in M9. GFP or YFP expression was analyzed using the 63× objective of a Zeiss Axio Imager D1 microscope and a Zeiss AxioCam MRm camera.

Two different *lin-12*-rescuing arrays, *arEx576* and *arEx1442*, are used in this study. *arEx576* was made by coinjecting GS#p101i [*lin-12p*::*lin-12(+)*] at 5 ng/μL, *unc-122p*::*gfp* at 10 ng/μL, and *mir-84p*::*2xnls-yfp* polymerase chain reaction product at 15 ng/μL. It expresses the coinjection markers strongly and rescues the sterility and AC/VU defects of *lin-12(0)*. MS-loss mosaics are easily identified by loss of *unc-122*p::GFP expression in coelomocytes and by loss of *mir-84*p::2xNLS-YFP expression in the somatic gonad cells, including the DTCs and ventral uterine cells. *arEx1442* was made by coinjecting linearized DNA (GS#p101i [*lin-12p*::*lin-12(+)*] and *myo-3*p::*mCherry* at 1 ng/μL and N2gDNA at 50 ng/μL) into *lin-12(n941)/arIs131unc-32(e189)* hermaphrodites and isolating lines that rescue the Pvl and sterility defects of *lin-12(0)* homozygotes.

*arIs208* was made by coinjecting linearized pMS66 [*hlh-2*p::*2xnls-tagRFP*], pMH86 [*dpy-20*(*+*)], and *ttx-3*p::*gfp* at 1 ng/μL, and *Pvu*II-digested N2 genomic DNA at 50ng/μL, into 20 P0 hermaphrodites of CB1282 (*dpy-20[e1282ts]*). Non-Dpy worms were isolated to establish lines, and the brightest extrachromosomal line was integrated as described (Mello and Fire 1995). The *arIs208* integrant was outcrossed ten times to N2 to make GS6793, which was then used for other strain constructions.

### Assessing AC number in *lin-12(0)* mutants

To assess maternal *lin-12* activity, homozygous *lin-12(0)* segregants were identified from *lin-12(0)/hT2[qIs48]* parents by loss of the pharyngeal GFP marking the balancer. The balanced strains also contained an AC marker. The number of ACs was scored by counting the number of bright GFP+ cells, as marked by *cdh-3*p::GFP *(arIs51)*. *lin-12(0)* homozygotes were grown at 20° by picking a large number of *lin-12(0)* hermaphrodites (lacking the balancer) and allowing them to establish a population from rare fertile individuals. Establishing this population therefore took several generations, but it allowed us to examine *lin-12(0)* progeny without any maternal *lin-12* contribution.

To assess temperature-sensitivity, homozygous *lin-12(0)* segregants were identified from parents that carried a GFP-marked rescuing extrachromosomal array. Gravid *lin-12(0)*; *arEx576*[*lin-12(+)*] were placed at either 20° or 25°. Progeny lacking *arEx576* were scored 2 d later using the 63× objective of a Zeiss Axio Imager D1 microscope with a Zeiss AxioCam MRm camera. The number of ACs was scored in mid-L3 to early-L4-stage hermaphrodites, as assessed by vulval development (Pn.px to early invagination). Cells with bright expression of the AC markers *arIs222* and *arIs51* were considered to be ACs.

We note that the α cells, Z1.ppp and Z4.aaa, always become ACs in *lin-12(0)* (Seydoux *et al.* 1990). We did not observe any ACs dividing, and no more than four ACs were observed in any individual. Thus, we interpret the additional ACs in *lin-12(0)* mutants to be the result of β cells adopting the AC fate and not the α cells dividing to yield additional ACs. Significant differences were determined with two-tailed Fisher’s exact tests (GraphPad software, quickCalcs 2 × 2 contingency table), comparing worms grown at 20° *vs.* 25°.

### Obtaining and scoring genetic mosaics lacking *lin-12* and *glp-1* in the somatic gonad

*lin-12(0) glp-1(0)* mutants arrest in the L1 stage (Lambie and Kimble 1991). Thus, mosaic animals were identified as segregants from *lin-12(0) glp-1(0)/ hT2[qIs48]*; *arEx576* by the absence of pharyngeal GFP marking the balancer *hT2[qIs48]* in addition to loss of the extrachromosomal array in the MS lineage. The MS lineage gives rise to all somatic gonad cells and to the six coelomocytes. We scanned plates for individuals lacking the *hT2* balancer, indicating *lin-12(0) glp-1(0)* homozygotes, and lacking GFP expression in all six coelomocytes (*unc-122*p::GFP), indicating a mosaic with a loss of *arEx576* in MS. Once the mosaics-of-interest were identified, we waited until *cdh-3*p::GFP was visible under the dissecting scope and then mounted the worms on a slide to score the number of ACs. At this step, we also verified that the *lin-12(+)* array was lost in the gonad by checking for loss of *mir-84*p::YFP expression. All three MS mosaic animals had only 2ACs. We estimate that approximately 2000 larvae were scanned to identify the three MS-loss mosaics.

### Scoring GFP-POP-1 reporter expression

All strains for this set of experiments were grown at 25°. We marked α and β cells in red (*arIs208* and *arIs222*) to facilitate scoring GFP-POP-1 at a developmental stage based on the size of the gonad and on the size and position of the α and β cells. This stage was chosen to be analogous to the stage at which GFP-POP-1 begins to accumulate asymmetrically in the daughters of Z1 and Z4, as this asymmetry develops after their birth (Siegfried *et al.* 2004).

The four α and β cells are a similar small size when born. Soon after, the β cells become larger than the α cells, which remain small during the AC/VU decision. We examined L2 larvae in which the gonad size remained small (prior to elongation), but the difference in size between α and β cells was becoming apparent. We mounted worms of this stage and looked for visible differences in expression level between α and β cells. For *qIs74*; *arIs208*, we used a Zeiss Cell Observer Z1 SD with a Photometrics Evolve EMCCD camera, and for strains containing *hlh-2*prox::GFP and GFP-POP-1 transgenes, we used a Zeiss Axio Imager D1 microscope with a Zeiss AxioCam MRm camera.

### Data availability

All strains, plasmids, and primer and sequence files are available upon request.

## Results

### *lin-12* activity contributes to the robustness of βVU fate at elevated temperature

**Figure 2:**
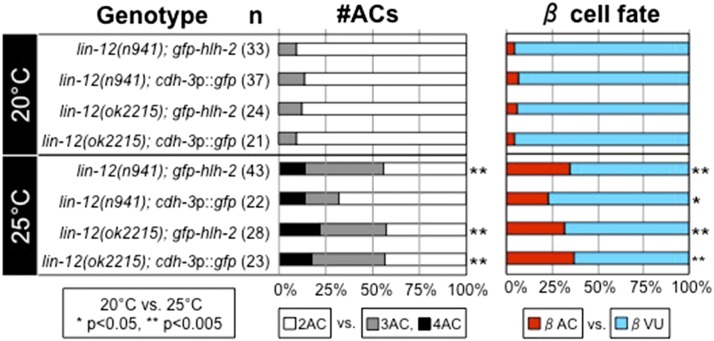
The *lin-12* null phenotype is heat-sensitive. *lin-12(0)* hermaphrodites are sterile. *lin-12(0)* progeny carrying an AC marker were identified (see the *Materials and Methods*) and scored for the number of ACs (#ACs, left graph, in gray-tone); also shown is the proportion of β cells that become ACs instead of VUs (β cell fate, right graph, in color) at 20° and 25°. Two-tailed Fisher’s exact tests were used to assess the differences in the number of ACs (2AC *vs.* >2AC) and in the fate of the β cells (AC *vs.* VU) of each strain at 20° *vs.* 25°.

At 20°, in mutants homozygous for the reference null allele *lin-12(n941)*, both α cells always adopt the AC fate; only 5% of β cells do, so that 90% of *lin-12(n941)* hermaphrodites have 2 ACs and 10% have 3 ACs [(Greenwald *et al.* 1983; Seydoux *et al.* 1990); see Figure 2, left graph, in gray-tone]. However, the proportion of animals with >2 ACs, and therefore the percentage of β cells that become ACs (Figure 2, right graph, in color), increases at 25°: about 50% of *lin-12(n941)* hermaphrodites have three or even four ACs (Figure 2, left graph). These observations suggest that *lin-12* activity contributes to robustness of β−cell specification and fate commitment at elevated temperature, even though *lin-12* activity is not absolutely required for a β cell to adopt the VU fate.

Importantly, the increase in the number of βACs only becomes apparent in mid-L3 or later larvae; in early L3 animals (before the VPCs divide), the majority of *lin-12(n941)* larvae only have two committed ACs, even at 25°: 86% 2AC (18/21) and 14% 3AC (3/21). This observation is consistent with laser ablation studies showing that the β cells in a *lin-12(0)* maintain their potential to be ACs even after the α cells have differentiated as ACs (Seydoux *et al.* 1990).

*lin-12(n941)* is associated with an amber codon in the fifth EGF-like motif of the ectodomain, leading to a potential protein that terminates after amino acid W400 (Wen and Greenwald 1999). Because combining this allele with a mutation in *smg-1* results in embryonic lethality, we infer that the message is normally subject to nonsense-mediated decay and that embryonic lethality likely results from a deleterious effect of the truncated LIN-12(1-400) protein produced when the mRNA is stabilized (Wilkinson 1994). To test whether the temperature-sensitive defect in VU fate is specific to *lin-12(n941)*, we obtained a deletion allele *lin-12(ok2215)* (Figure 1C), and examined the number of ACs in both mutants at 20°, and 25° (Figure 2). The deletion allele and the reference null allele both display similar temperature-sensitivity, with additional ACs being specified at the higher temperature. These results suggest that *lin-12* activity, which is absolutely required for the AC/VU decision by the α cells at all temperatures, contributes to the robustness of the βVU fate, and that the process by which the α and β cells become different is inherently temperature-sensitive.

In the next section, we address the question as to how the βVU fate is specified in *lin-12(0)* mutants by asking if there is another source of *Notch* activity and in the subsequent two sections, we look at the potential contribution of the Wnt/β-catenin asymmetry pathway nuclear effector POP-1.

### Maternal *lin-12*/Notch activity and zygotic *glp-1*/Notch activity do not influence βVU fate

As *lin-12(0)* hermaphrodites have a highly penetrant sterility defect, most previous studies have examined *lin-12(0)* animals segregating from heterozygous mothers, or, more recently, germline loss of extrachromosomal arrays. However, rarely, *lin-12(0)* homozygous hermaphrodites can produce a few progeny. If maternal activity specifies the βVU fate, we would expect such progeny to have four ACs. However, we found that 10 of 11 offspring of homozygous *lin-12(0)* hermaphrodites had two ACs and 1 of 11 had three ACs at 20°, similar to the proportion obtained from heterozygous parents (Figure 2, data not shown). Thus, maternal *lin-12* activity does not account for the βVU fate observed in *lin-12(0)* hermaphrodites segregating from heterozygous mothers.

There is a second *C. elegans Notch* gene, *glp-1* (Yochem and Greenwald 1989). Maternal *glp-1* activity mediates cell−cell interactions in the early embryo; zygotic *glp-1* activity mediates later embryonic interactions and proliferation of the germline (Austin and Kimble 1987; Priess 2005; Priess *et al.* 1987). Zygotic *glp-1* activity is also functionally redundant with *lin-12* in several late embryonic cell fate decisions (Lambie and Kimble 1991), and GLP-1 can functionally substitute for LIN-12 in the AC/VU decision when expressed under the control of *lin-12* regulatory sequences (Fitzgerald and Greenwald 1995). Thus, if zygotic *glp-1* activity is present in the β cells, it might promote the βVU fate.

**Figure 3:**
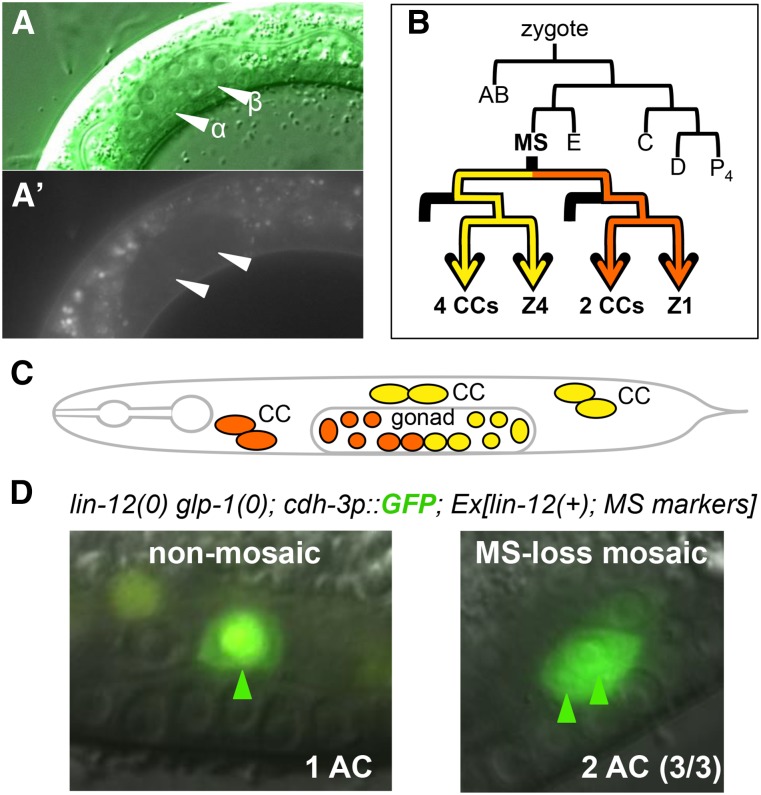
Expression and mosaic analysis indicate that *glp-1* does not specify the βVU fate. (A) GLP-1-GFP fosmid reporters are not expressed at the time that the α and β fates are specified. Photomicrographs of strain GS6433. (B) The MS lineage showing the lineal origin of the six coelomocytes (CC), Z1, and Z4. (C) Schematic view of the relevant cells for mosaic analysis. (D) Genetic mosaics lacking both *lin-12* and *glp-1* in the MS lineage have 2 ACs, like most *lin-12(0)* hermaphrodites, rather than 4ACs, the predicted result if *glp-1* played a redundant role with *lin-12* in specifying βVU fate.

We first asked whether GLP-1 is expressed in the somatic gonad by examining GLP-1-GFP translational fosmid reporters (see the section *Materials and Methods*). We did not observe GFP in the α or β cells (or any other somatic gonadal cells in the L2 stage), suggesting that GLP-1 is not present at the time the βVU fate is specified (Figure 3A).

We next asked whether there is a functional role for *glp-1* in the somatic gonad. *glp-1(0)* hermaphrodites can only be obtained as segregants from heterozygous mothers, because maternal *glp-1* activity is necessary for essential early embryonic cell fate decisions; these segregants are sterile, because *glp-1* activity is required for germline development but have normal somatic gonad development and vulval induction (Austin and Kimble 1987; Priess *et al.* 1987). Animals lacking the activity of both *lin-12* and *glp-1* arrest as L1 larvae due to defects in the excretory system and rectum, both derived from the AB lineage (Lambie and Kimble 1991). The somatic gonad is derived from the MS lineage, so we were able to obtain viable genetic mosaics lacking both *lin-12* and *glp-1* activity in the somatic gonad by loss of a rescuing extrachromosomal array in MS (Figure 3, B−D). The three mosaics we obtained had two ACs, as in *lin-12(0)* alone, rather than four ACs as would be expected if the two *Notch* genes function redundantly to specify βVU fate (Figure 3D). Together, the expression and genetic analyses indicate that *glp-1* does not contribute to the βVU fate.

### Asymmetric distribution of POP-1 suggests that the Wnt/β-catenin asymmetry pathway may act positively in β cells

Wnt signaling is an important pathway for generating differences between sister cells in *C. elegans* [reviewed in (Sawa and Korswagen 2013)]. There is no single “Wnt pathway,” but in canonical pathways, differential activation of Wnt signal transduction can lead to differences in the level of POP-1, the sole *C. elegans* ortholog of TCF, the nuclear effector of Wnt signal transduction.

The Wnt/β-catenin asymmetry pathway, a variant canonical pathway, acts in the first division of Z1 and Z4, the somatic gonad precursor cells, to establish a proximal-distal axis such that Z1.a and Z4.p have a distal identity, and Z1.p and Z4.a have a proximal identity (Siegfried *et al.* 2004; Siegfried and Kimble 2002). The phenotypes resulting from *pop-1* mutants in a *lin-12(0)* background suggest that subsequent divisions in the Z1 and Z4 lineages also may involve the Wnt/β-catenin asymmetry pathway (Siegfried and Kimble 2002), but alterations in the number of ACs resulting from earlier effects in the lineage make it difficult to draw conclusions about the role in the α and β cells *per se*.

**Figure 4:**
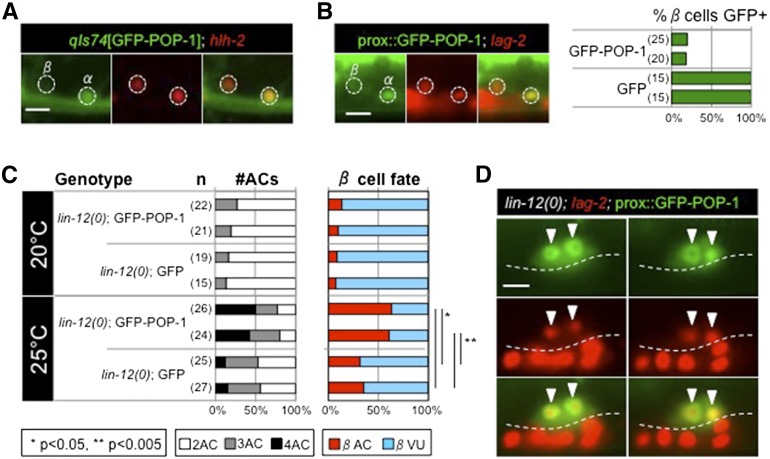
POP-1 shows asymmetric expression in the α and β cells, and can promote AC fate. (A) The canonical GFP-POP-1 reporter *qIs74* (Siegfried *et al.* 2004) shows brighter expression in the α cells than in β cells (white dotted circles, cells marked in red with *arIs208*) in 17/18 animals observed. (B) Two *hlh-2*prox::GFP-POP-1 transgenes, which express GFP-POP-1 specifically in the α and β cells (marked in red by *arIs222*), show fully penetrant GFP expression in the α cells but expression is frequently weak or undetectable in β cells (right). By contrast, two *hlh-2*prox::GFP transgenes are expressed equally in both α and β cells. A representative image with *arEx2220* [*hlh-2*prox::GFP-POP-1] is shown (left). (C) Expression of GFP-POP-1 from *hlh*-2prox promotes the βAC fate in *lin-12(0)* larvae at 25°. *lin-12(0)* null hermaphrodites carrying two independent *hlh*-2prox::GFP-POP-1 or *hlh*-2prox::GFP transgenes were raised at 20° or 25°. The number of ACs was assessed with *arIs222* [*lag-2*p::2xNLS-TagRFP]. Each line in which GFP-POP-1 is expressed shows a significant difference in the proportion of β cells adopting the AC fate at 20° *vs.* 25° by two-tailed Fisher’s exact test (*P < 0.05, **P < 0.005), as compared to lines in which GFP alone is expressed. In addition, each GFP-POP-1 line shows a significant difference in the number of ACs at 25°C *vs.* 20°C by two-tailed Fisher’s exact test (*P* < 0.001). The number of worms scored is shown in parentheses. Full genotype: *lin-12(n941) pha-1(e2123ts)*; *arEx*[prox::GFP or GFP-POP-1], after losing the *lin-12*-rescuing array *arEx1442*. (D) A representative *lin-12(0)*; *arEx2219*[*hlh*-2prox::GFP-POP-1] L3 hermaphrodite raised at 25°C has four ACs, shown in two planes of focus (left, right). Arrowheads indicate the ACs and *lag-2* marks the ACs in red (with vulval cells also marked, below the dotted line). Scale bars are 5μm.

In many different sets of sister cells, including the distal daughters of Z1 and Z4, nuclear levels of the effector POP-1 are lower in sister cells with greater Wnt signal-transduction activity (Bertrand and Hobert 2009; Herman 2001; Lin *et al.* 1995; Phillips *et al.* 2007; Phillips and Kimble 2009; Rocheleau *et al.* 1997; Thorpe *et al.* 1997; Siegfried *et al.* 2004). Thus, to explore the possibility that Wnt signal-transduction activity generates the asymmetry between α and β cells that allows *lin-12*-independent βVU fate, we looked for evidence of asymmetry in the accumulation of POP-1. We first used *qIs74*, an established translational reporter (Siegfried *et al.* 2004), and observed that GFP-POP-1 displays a dynamic pattern: accumulation is initially similar in α and β cells, and then α cells accumulate more nuclear GFP-POP-1 than β cells (Figure 4A).

We then created two independent transgenic lines expressing GFP-POP-1 under the control of *hlh-2*prox, a promoter that is specific to Z1.pp, Z4.aa and their daughters, the α and β cells (Sallee and Greenwald 2015). In the L2 stage, *hlh-2*prox drives expression of GFP in all four cells; initially, before specification, the level of expression is similar in the unspecified α and β cells but later becomes relatively greater in the AC and αVU than in the βVUs (Sallee and Greenwald 2015). This initial stage can be recognized by morphological criteria (see *Materials and Methods*). We observed that, at the initial stage, the α cells accumulate more nuclear GFP-POP-1 than the β cells (Figure 4B), suggesting that activation of Wnt signal transduction in the β cells may contribute to the difference in AC potential between the α and β cells.

### Increasing POP-1 activity promotes the AC fate of β cells in the absence of *lin-12* activity

To ask whether the lower level of POP-1 in β cells (relative to α cells) is important to promote their specification as VUs instead of ACs, we sought to increase *pop-1* level or activity in the developing ventral uterus by using the *hlh-2*prox::GFP-POP-1 transgenes described above to augment endogenous *pop-1* activity. In a *lin-12(0)* background, we found that *hlh-2*prox::GFP-POP-1 transgenes resulted in a significantly greater proportion of β cells adopting the AC fate than in control strains carrying *hlh-2*prox::GFP transgenes (Figure 4, C and D). These observations suggest that elevated *pop-1* activity in β cells promotes the AC fate, and therefore that the lower POP-1 level normally observed in the β cells may promote or permit their VU fate. Furthermore, because the *hlh-2*prox::GFP-POP-1 transgenes do not cause β cells to adopt the AC fate in a *lin-12(+)* background, we infer that the endogenous *lin-12* activity in β cells counters *pop-1* to promote the VU fate.

## Discussion

During development of the hermaphrodite somatic gonad primordium, two α cells, derived from different somatic gonad founder cells, initially have the potential to be an AC, as do their sisters, the β cells. However, the β cells lose that potential rapidly, whereas the α cells maintain that potential until *lin-12*/Notch signaling between them resolves their fates. In the end, the somatic gonad primordium contains a single AC, derived from one of the α cells, and three VUs, one derived from the other α cell and two derived from the β cells. Our results have illuminated aspects of how this hierarchy of developmental potential between the α and β cells is generated.

### Robustness of ventral uterine fate specification

We found that *lin-12*/Notch plays a role in ensuring the robustness of the β cell VU fate under elevated temperature conditions. Robustness is a critical property of developing systems, ensuring a correct outcome under environmental perturbations. Since the AC is critical to induce the vulva, and perturbations in the number of ACs can alter vulval development, it is not surprising to find that there are robust mechanisms to ensure its proper specification.

Moreover, robustness has been proposed to be a prerequisite for evolving, complex dynamic systems (Kitano 2004), allowing cryptic genetic change to accumulate without a change in developmental output (Félix and Wagner 2008). The specification of the AC is an example of such an evolving, dynamic process, as many variations in the specification of the AC exist in other nematode species: although in *C. elegans* the α cells appear to have equal potential to be the AC, in other species, AC fate can be specified in a biased or in a fully fixed manner in the Z1 or Z4 lineage, and AC potential can be retained or lost within these lineages. Despite these differences, the ultimate outcome—one AC and three VUs—remains the same (Félix and Sternberg 1996).

### Wnt signaling and the difference in AC potential between α and β cells

We found that the α cells have greater nuclear POP-1 relative to the β cells and that augmenting *pop-1* activity in the absence of *lin-12* activity increases the proportion of β cells adopting the AC fate rather than the VU fate. Thus, Wnt signal transduction activity in the β cells may contribute to their early loss of AC competence relative to the α cells and the robustness of the βVU fate in the absence of *lin-12*.

There is no single “Wnt pathway”; instead, there are several distinct signal transduction mechanisms. In “canonical” pathways, Wnt signaling is mediated by POP-1/TCF and β-catenin, which acts as a transcriptional coactivator for TCF. In the standard canonical pathway in *C. elegans*, stabilization of BAR-1/β-catenin and its translocation to the nucleus lead to activation of target genes [reviewed in (Sawa and Korswagen 2013)]. In the “Wnt/β-catenin asymmetry pathway,” a divergent canonical pathway, reducing the level of POP-1 favors the formation of the activation complex with the divergent β-catenin SYS-1 (Kidd *et al.* 2005). The differential accumulation of nuclear POP-1 and the genetic interaction we observed, that augmenting POP-1 promotes the AC fate of β cells, suggests that some form of a canonical pathway is involved.

The Wnt/β-catenin asymmetry pathway generates the difference in the potential of the daughters of Z1 and Z4 to generate the DTC, another specialized cell of the somatic gonad primordium, making it an attractive candidate for mediating the difference in AC potential between α and β cells. In the daughters of Z1 and Z4, SYS-1/β-catenin is initially present in similar levels but is preferentially degraded to generate a pattern of accumulation reciprocal pattern to POP-1 (Phillips *et al.* 2007). However, we could not reliably detect expression of SYS-1 in the α or β cells using the reporter that revealed differences in the Z1 and Z4 daughters, *qIs95* [*sys-1*p::VENUS-SYS-1] (data not shown). Thus, the pathway by which α and β cells become different remains an open question for future investigation.

### Wnt-POP-1/TCF and LIN-12/Notch signaling pathways lead to robust specification of ventral uterine cell fates

POP-1, like all TCF proteins, can function as a repressor or as an activator of transcription. In asymmetric divisions controlled by the Wnt/β-catenin asymmetry pathway, lower POP-1 resulting from Wnt signal transduction in one daughter is associated with activation of target genes whereas greater POP-1 in the other daughter is associated with repression of target genes (Sawa and Korswagen 2013).

**Figure 5:**
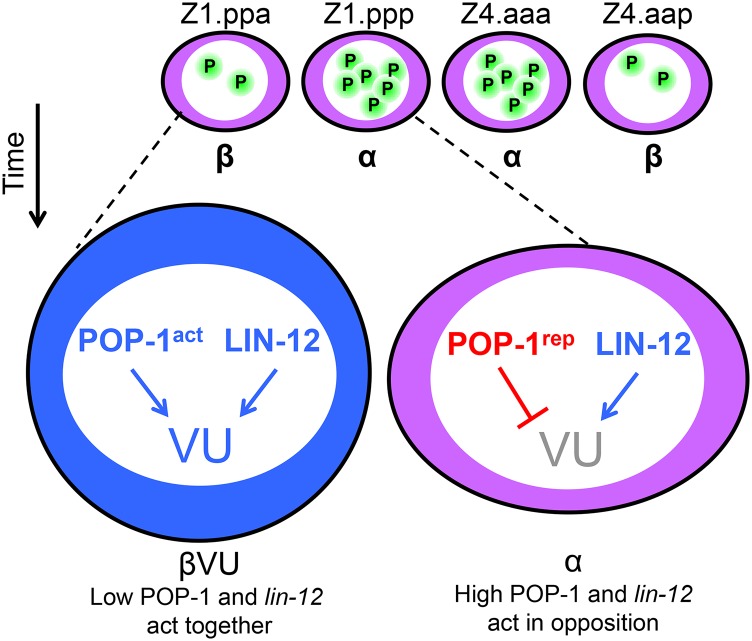
Model: the difference in levels of POP-1 establishes or maintains the difference between α and β cells. Our data suggest that POP-1 and LIN-12 work together in β cells and in opposition in α cells to pattern the ventral uterus. It is possible, but not essential for this model, that these two transcription factors converge on one or more common targets. LIN-12 functions as an activator of target gene expression and promotes the VU fate (Greenwald and Kovall 2013). The level of POP-1 (green) differ between α and β cells (see Figure 4). Low levels of POP-1 have been associated with its function as an activator of transcription and high levels are associated with its function as a repressor of transcription (Phillips and Kimble 2009). Thus, in β cells, low POP-1 may act with LIN-12 to promote the transcription of common targets that favor or execute VU fate. In the α cells, high POP-1 acts as a transcriptional repressor, where it blocks VU fate and acts in opposition to LIN-12. Thus, high POP-1 may directly oppose LIN-12, raising the threshold for specification of the αVU fate.

In view of the relationship between asymmetric distribution of POP-1 and its mechanism of action in other paradigms, our results lead us to propose a model (Figure 5) in which (i) the relatively high level of POP-1 in α cells favors the repressor mode and represses target genes that oppose AC potential, thereby keeping α cells competent to generate the AC, and (ii) the relatively low level of POP-1 in β cells favors the activator mode (β-catenin bound) and activates targets that restrict their AC-competence. The direct transcriptional targets of POP-1 in its different modes may be the same, distinct, or overlapping.

We also propose that LIN-12/Notch and POP-1 in its activator mode work together in β cells to promote robust specification of VU fate. An intriguing possibility is that they function additively or redundantly to promote degradation of HLH-2, a pro-AC factor, in β cells to restrict their AC potential rapidly (Karp and Greenwald 2003, 2004; Sallee and Greenwald 2015). Future identification of targets of LIN-12 and POP-1 in ventral uterine development will be important for elucidating the mechanism by which AC potential and its relationship to cell fate specification is governed in α and β cells.
